# Integration of Transcriptome Resequencing and Quantitative Proteomics Analyses of Collagenase VII-Induced Intracerebral Hemorrhage in Mice

**DOI:** 10.3389/fgene.2020.551065

**Published:** 2020-12-17

**Authors:** Fang Cao, Yu Guo, Qiang Zhang, Yinchun Fan, Qian Liu, Jiancheng Song, Hua Zhong, Shengtao Yao

**Affiliations:** ^1^Department of Cerebrovascular Disease, Affiliated Hospital of Zunyi Medical University, Zunyi, China; ^2^Department of Radiology, Daping Hospital, Army Medical University, Chongqing, China; ^3^College of Life Sciences, Wuhan University, Wuhan, China

**Keywords:** intracerebral hemorrhage, transcriptome resequencing, proteomic analyses, inflammation, competing endogenous RNA, protein–protein interactions

## Abstract

**Objective:**

Intracerebral hemorrhage (ICH) is a subtype of stroke with high mortality and morbidity rates. Our aim was to comprehensively analyze transcriptome and proteome in an experimental ICH model.

**Methods:**

All mice were divided into ICH model (*n* = 3) and sham groups (*n* = 3). ICH was induced by collagenase VII. The ipsilateral hemisphere was used for whole transcriptome and proteomics resequencing. After preprocessing, differentially expressed lncRNAs (DElncRNAs), mRNAs (DEmRNAs), miRNAs (DEmiRNAs), and DEproteins between ICH and sham groups were identified. Functional enrichment analysis was performed using the clusterProfiler package, followed by protein–protein interaction (PPI) analysis. After that, the Pearson correlation coefficient between DEmRNAs and DElncRNAs or between DEmRNAs and DEproteins was calculated. DElncRNAs with similar functions were analyzed by the GOSemSim package. After prediction of DEmiRNA–DEmRNA and DElncRNA–DEmiRNA relationships, a competing endogenous RNA (ceRNA) network was constructed. Several DEmRNAs and DElncRNAs were validated in ipsilateral hemisphere tissues of the ICH model and control groups using RT-qPCR and western blot.

**Results:**

Between the ICH and sham groups, 31 DElncRNAs, 367 DEmRNAs, 35 DEmiRNAs, and 96 DEproteins were identified. DEmRNAs were mainly enriched in inflammation, such as cytokine–cytokine receptor interaction, IL-17, and TNF signaling pathways. A PPI network of DEmRNAs was constructed and hub genes were identified, such as IL6 (degree = 59), TNF (degree = 44), and CXCR2 (degree = 39). 24 DElncRNAs with similar functions were identified, including 15 up- and 9 down-regulated lncRNAs. After integration of DEmiRNA–DEmRNA and DElncRNA–DEmiRNA relationships, we constructed a ceRNA network, composed of 71 DEmRNAs, 17 DEmiRNAs, and 12 DElncRNAs. RT-qPCR and western blot results confirmed that C3, Fga, and Slc4a1 proteins were more lowly expressed and Penk was more highly expressed in ICH than control groups, which could become potential markers for ICH.

**Conclusion:**

Our findings identified ICH-related DE-RNAs and proteins and potential molecular mechanisms of ICH by transcriptome resequencing and quantitative proteomic analyses.

## Introduction

Strokes are divided into either ischemic stroke or ICH. ICH accounts for about 15% to 20% of all stroke cases, characterized by hematoma expansion and inflammation. ICH patients have a mortality rate of up to 40% in the first month. The mortality and disability rates of ICH patients is much higher than that of ischemic stroke patients (Fang et al., 2013). Although efforts have been made to reduce ICH and post-ICH complications, the clinical outcomes have been suboptimal. About 20% of ICH survival patients suffer from neurological dysfunction (Duan et al., 2016). However, effective treatment options for ICH are still lacking, and few studies provide evidence to guide ICH treatment (Jia et al., 2018). ICH patients’ poor prognosis is closely related to the complicated pathogenesis of ICH. A previous study has shown that targeting ICH-related molecules could be a promising therapeutic strategy (Liu et al., 2019). Thus, it is urgent to explore and understand the molecular mechanisms of ICH.

Non-coding RNAs (ncRNAs), such as lncRNA and miRNA, exhibit important biological functions (Beermann et al., 2016; Matsui and Corey, 2017). ncRNAs could affect the expression of target genes. lncRNA, a non-coding RNA larger than 200 nucleotides, has been reported to play an important role in the pathophysiology of ICH (Zhang and Wang, 2019). Furthermore, lncRNA as a sponge of miRNA can indirectly regulate the expression of downstream messenger RNA (mRNA), which is described as ceRNA (Park et al., 2018). The interactions between genes or proteins are involved in the pathogenesis of diseases. The functions of mRNA, miRNA, lncRNA, and proteins in ICH remains largely unknown.

Herein, this study comprehensively analyzed the transcriptome and proteome of ICH based on collagenase VII-induced ICH mouse models, which could provide an insight into ICH-related DE-RNAs, proteins, and potential molecular mechanisms.

## Materials and Methods

### Animals

Male C57BL/6 mice (age: 10–12 weeks; weight: 22–25 g) were purchased from the Animal Institute of the Third Military Medical University. All mice were fed under a 12 h light/dark cycle in a temperature-controlled and specific pathogen-free environment. All animal experiments conformed to the animal experiment manual approved by the Animal Ethics Committee of Zunyi Medical University. All experiments were performed and reported according to the Animal Research: Reporting *in vivo* Experiments (ARRIVE) guidelines.

### ICH Mouse Model

The collagenase VII-induced ICH model was established as previously reported (Xie et al., 2016). All mice were randomly divided into ICH model group (*n* = 3) and sham operation (*n* = 3). All mice were anesthetized by intraperitoneal injection of pentobarbital sodium and placed on a brain stereotaxic apparatus (RWD, China) in the ventricumbent position. A previously reported coordinate point (coordinates: 0.2 mm anterior, 2.3 mm lateral, and 3.5 mm depth to bregma) was utilized to inject 1 μL bacterial collagenase (0.0375 units per 1 μL, type VII-S; Sigma-Aldrich, United States) into the striatum at a rate of approximately 0.1 μL/min using a microinjection pump (Longer, TJ-2A/L0107-2A, China). After that, the needle was kept at the injection point for 5 min to prevent liquid backflow. The microsyringe was removed slowly and the cranial pinhole was closed with bone wax. The incisions in the skin were sutured. The sham operation group was injected with an equivalent volume of PBS only, and the other operations were the same.

### Mice Hemisphere Harvest

In this study, all mice were scanned by a Bruker 7T MRI (70/20) system (BrukerBiospin, Billerica, MA, United States) (Cao et al., 2016). After the rotarod test pre- and post-CCI, mice were anesthetized with gas mixture (induction: 5% isoflurane with 1 L/min O_2_, maintenance: 1% isoflurane with 1 L/min O_2_), mounted in a Bruker animal bed, and their body temperature was maintained at 37°C with respiratory rate continuously monitored. T2-weighted images were acquired using RARE (TR = 4000, TE = 45, RARE factor 8, 0.5 mm, FOV 2.5 cm, 256 × 256). Images were analyzed using Bruker ParaVision 6.0 software. The lesion volumes were determined as pixels that had T2 values higher than the mean plus two standard deviations of the value in the homologous contralesional region. The model was confirmed to be successfully constructed before euthanizing at 24 h after ICH injury or sham injury, and the whole brain was divided into two halves, as described in a previous study (Cao et al., 2016). The ipsilateral hemisphere was used for whole transcriptome resequencing by Novaseq 6000 (Illumina, United States) and whole proteomics resequencing that were analyzed on an Orbitrap Fusion Lumos Tribrid mass spectrometer (Thermo Scientific, United States).

### Whole Transcriptome Resequencing Analysis

Total RNA was extracted from tissues using TRIzol reagent (Invitrogen, United States). Standard denaturing gel electrophoresis was used to assess RNA integrity. Extracted RNA was transcribed into cDNA. Mouse reference genome and annotation information were downloaded using Ensembl Genome Browser [version: GRCm38.p6 (GCA_000001635.8)]. An index of the reference genome was created via hisat2. The transcriptome sequencing double-end data were firstly cleaned using Trim Galore. Trim Galore can automatically identify and remove the 3′ end adapter. In this way, the transcriptome data were quantified after obtaining clean data from raw data. Two separate library preparations were used for the long and small RNAs. The ribosomal depletion was utilized for library preparations of the long RNAs. Furthermore, small RNA-seq cDNA library preparation was performed for the small RNAs.

The quantification of the RNA (including lncRNA and mRNA) transcriptome was performed using hisat2. The parameters were defaulted. The alignment rates were as follows: M1: 96.65% overall alignment rate, M2: 96.61% overall alignment rate, M3: 96.63% overall alignment rate, M7: 96.47% overall alignment rate, M8: 96.86% overall alignment rate, and M9: 96.52% overall alignment rate. Among them, M1-3 represented three ICH mouse models and M7-9 represented three sham operation mice. After sorting the mapping results by samtools, featureCounts was used to quantify. The quantitative results of the RNA transcriptome data were obtained for further analysis.

The miRNA transcriptome was quantified using miRDeep2. All mouse miRNA sequence data were downloaded as alignment references and as annotated files from the miRBase database (version: 22) (Griffiths-Jones et al., 2006). Trim Galore was used to remove the adapters in the raw data, and then cutadapt was used to screen sequences with a sequence length of 18–25. The above result was used as a clean read for subsequent alignment analysis. Redundant sequences were removed by miRDeep2 and unique reads were obtained. Based on mouse miRNA data in the miRBase database, quantification of miRNA transcriptome data was presented.

### Differential Expression Analysis

Differential expression analysis was performed by the likelihood ratio test method of the edgeR package (Robinson et al., 2010). After deletion of genes with a lower abundance and normalization by TMM, DElncRNAs, DEmRNAs, and DEmiRNAs between the ICH and sham groups were identified, and the threshold was set to FDR (adjusted *p*-value) < 0.05 and log2| fold change (FC)| > 1.5. Finally, the results were visualized as volcano plots and heat maps.

### Functional Enrichment Analysis

Functional enrichment analysis was performed using the clusterProfiler package, including GO and KEGG pathway (Yu et al., 2012). GO contains cellular component (GO-CC), molecular function (GO-MF), and biological process (GO-BP). P-value < 0.05 after correction was considered significantly enriched.

### PPI

DEmRNAs were imported into the STRING database for PPI analysis. The combined score > 0.7 was set as the threshold. The PPI network was constructed using the Cytoscape. CytoNCA plug-in of Cytoscape was used to calculate degree centrality, betweenness centrality, and closeness centrality. Nodes with high scores were considered as hub genes.

### Correlation Analysis of lncRNAs and mRNAs

For the DEmRNAs and DElncRNAs obtained, the correlation between lncRNA and mRNA was calculated using the Pearson correlation coefficient. *P*-value was corrected by the BH method and corrected *p*-value < 0.01 indicated that there could be a significant correlation between lncRNA and mRNA. The lncRNA–mRNA network was drawn through the Cytoscape.

### lncRNA Functional Similarity Analysis

To explore potential functions of DElncRNAs, functional enrichment analysis of target genes of DElncRNAs was performed. The semantic similarity between GO-BP terms enriched by target genes of DElncRNAs was quantified using the Resnik method and Wang method provided by GOSemSim package, which was used to measure the functional similarity between DElncRNAs (Wang et al., 2007; Yu et al., 2010).

### miRNA–mRNA Prediction and ceRNA Network Construction

For the obtained DEmiRNAs, miRNA-target prediction was performed by miRWalk, Microt4, miRanda, mirbridge, miRDB, miRMap, miRNAMap, Pictar2, PITA RNA22, RNAhybrid, and Targetscan databases on miRWalk 2.0 platform. Prediction results supported by over seven databases were considered reliable miRNA–mRNA relationships.

After obtaining the sequence of the DElncRNAs from the reference genome and the sequence of the DEmiRNAs from the miRBase database, the miRanda tool (parameters: -sc 120, -en -20) was used to predict whether there was a binding site between miRNA and lncRNA. If there were more than five binding sites, it was considered that there was a regulatory relationship between miRNA and lncRNA.

After integration of DEmiRNA–DEmRNA and DElncRNA–DEmiRNA relationships, a lncRNA–miRNA–mRNA ceRNA network was constructed.

### Proteomic Analysis

Tissues were lysed using RIPA lysis (Beyotime, Beijing, China). After centrifugation, the supernatant was collected. DEproteins were analyzed based on proteome resequencing analysis results, with the threshold of *q*-value < 0.05 and | log2FC| > 0.5. Functional enrichment analysis of these proteins was performed using the clusterProfiler package. Furthermore, a PPI network was conducted to predict the interactions between DEproteins.

### mRNA–Protein Correlation Analysis

Venn analysis was performed on DEmRNAs from transcriptome data and DEproteins from proteomic analysis. Pearson correlation coefficient was calculated to analyze the correlation between DEmRNAs and DEproteins.

### Real-Time Quantitative Polymerase Chain Reaction (RT-qPCR)

Total RNA was extracted from tissues by Trizol (Invitrogen, United States). The extracted RNA was reverse transcribed into cDNA in the following reaction procedures: 37°C for 60 min, 85°C for 5 min, and 4°C for 5 min. cDNA was amplified by SYBR Green PCR kit (#K0223; Thermo, Waltham, MA, United States) through the following procedures: 40 circles of 95°C for 10 min, 95°C for 15 s, 60°C for 1 min, 95°C for 15 s, and 60°C for 15 s. Primer sequences were as follows: Fga: 5′-GCG GCAGATGAGAATGGAG-3′ (forward), 5′-GTTCCCAGGACG CCAATAC-3′ (reverse); C3: 5′-TGGGAGAAGTTCGGCATA GAG-3′ (forward), 5′-GGTTGTTGAAGGCAGCATAGG-3′ (reverse); 5′-AGCACCAACAATGACGAAG-3′ (forward), 5′-TT CAGAACCGCATAAAGCC-3′ (reverse); Slc4a1: 5′-CAGGA CTACCCACTACAAC-3′ (forward), 5′-CCACCAGGACCATT ATCAG-3′ (reverse); GAPDH: 5′-CTGCCCAGAACATCAT CC-3′ (forward), 5′-CTCAGATGCCTGCTTCAC-3′ (reverse). The relative expression levels were quantified with the 2^–ΔΔCt^ method.

### Western Blot

Protein was extracted from tissues via RIPA (Beyotime, Beijing, China). The protein concentration was assessed by BCA kit (Beyotime). Then, protein samples were separated through SDS-PAGE, which was transferred onto PVDF membrane. The membrane was blocked by 0.5% skimmed milk for 2 h at room temperature, followed by incubation with primary antibodies against Protein Penk (1:1000; ab150346; Abcam, United States), C3 (1:1000; ab181147; Abcam), Fga (1:2000; ab108616; Abcam), Slc4a1 (1:1000; ab196798; Abcam), and β-actin (1:200; ab115777; Abcam) at 4°C overnight, and secondary antibodies (1:5000; ab7090) at room temperature for 2 h. Protein blots were analyzed through the Western Lighting Ultra (Thermo).

### Statistical Analysis

Statistical analyses were performed by R 3.6.3 and GraphPad 7.0. Data from experiments were presented as mean ± standard deviation. Paired student’s *t*-test was used for comparisons between the two groups. *P* < 0.05 was considered statistically significant.

## Results

### Identification of DElncRNAs and DEmRNAs for ICH

**FIGURE 1:**
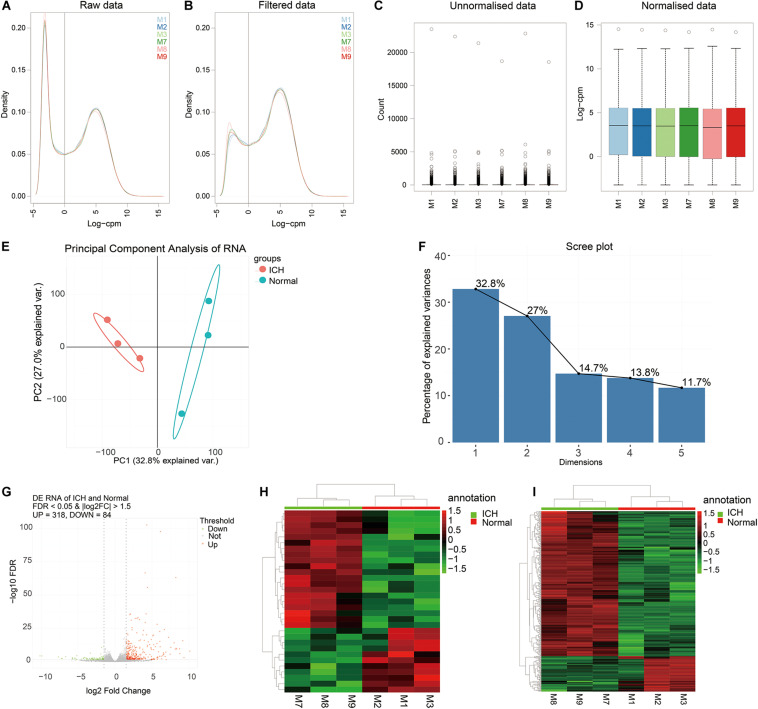
Identification of DElncRNAs and DEmRNAs for ICH. **(A,B)** The data density distribution curve before or after removing low-expressed lncRNAs or mRNAs. **(C,D)** The box plot showing the unnormalized data or normalized data by TMM. **(E)** PCA analysis of RNA transcriptome. **(F)** Scree plot of RNA transcriptome. **(G)** Volcano plot showing 318 up- and 84 down-regulated RNAs between ICH groups and sham groups. **(H)** Hierarchical clustering showing the differential expression patterns of DElncRNAs between ICH and sham groups. **(I)** Hierarchical clustering showing all DEmRNAs between ICH and sham groups. M1-3 represents three ICH mouse models and M7-9 represent three sham operation mice. Red indicates up-regulation and green indicates down-regulation.

In this study, we conducted ICH mouse models, and all the ICH mouse models were confirmed by the T2-weighted images from a Bruker 7T MRI (70/20) system (BrukerBiospin, Billerica, MA, United States) before use (Supplementary Figure S1). The ipsilateral hemisphere tissues of ICH model and sham groups were used for whole transcriptome resequencing analyses. Raw data were pre-processed and lowly expressed lncRNAs or mRNAs were removed (Figures 1A,B). Then, filtered data were normalized by TMM methods (Figures 1C,D). Before differential expression analysis, we performed PCA analysis. In Figure 1E, the PCA analysis results of the two dimensions (ICH and sham groups) showed that the interpretation of the data by the two dimensions exceeded 50% (32.8% + 27%). Beginning with comp. 2, the scree plot began to flatten, indicating that the two dimensions could well explain the characteristics of the data (Figure 1F). The above results suggested that there were obvious differences in the characteristics between the two groups of data. Thus, differential expression analysis results would be reliable. Differential expression analysis was performed using the edgeR package. As depicted in the volcano plot, there were 318 up- and 84 down-regulated RNAs between ICH groups and sham groups (Figure 1G). Among them, 31 DElncRNAs were identified, including 11 up- and 20 down-regulated lncRNAs (Figure 1H). Furthermore, there were 367 DEmRNAs between the ICH groups and sham groups, including 306 up- and 64 down-regulated mRNAs (Figure 1I). These DElncRNAs and DEmRNAs can accurately distinguish the two group samples into two clusters, indicating that the results of the differential expression analysis were reliable.

### Functional Enrichment Analysis and PPI Network of DEmRNAs

**FIGURE 2:**
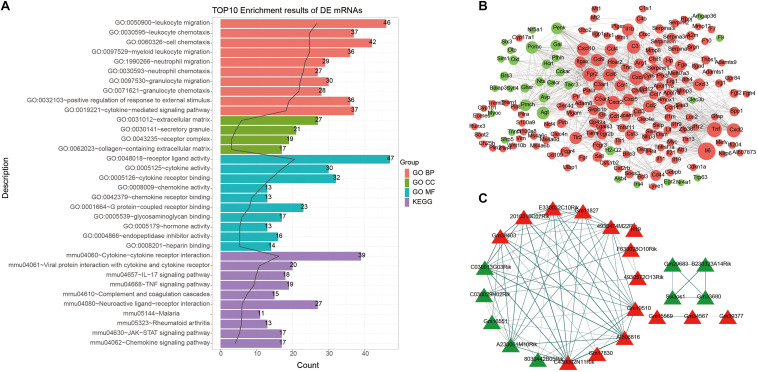
Functional enrichment analysis and PPI network of DEmRNAs. **(A)** The top 10 functional enrichment analysis results based on DEmRNAs. **(B)** A PPI network. The larger the circle, the greater the degree. **(C)** Functional similarity analysis of DElncRNAs. Red represents up-regulation and green represents down-regulation. The thickness of the line indicates the degree of functional similarity.

To explore potential functions of DEmRNAs, GO and KEGG functional enrichment analyses were performed. The top ten enrichment results were shown in Figure 2A. For GO enrichment analysis results, we found that these DEmRNAs were mainly enriched in inflammation responses, such as leukocyte, neutrophil, granulocyte, cytokine, and so on. As for KEGG pathway results, these DEmRNAs were also mainly enriched in immune-related pathways, including cytokine–cytokine receptor interaction, viral protein interaction with cytokine and cytokine receptor, IL-17 signaling pathway, TNF signaling pathway, complement and coagulation cascades, neuroactive ligand–receptor interaction, JAK-STAT signaling pathway, and chemokine signaling pathway.

A PPI network of DEmRNAs was constructed for ICH. There were 184 nodes, including 151 up- and 33 down-related genes (Figure 2B). The top ten nodes with the highest degree were as follows: IL6 (degree = 59), TNF (degree = 44), CXCR2 (degree = 39), CXCL1 (degree = 39), CXCL2 (degree = 39), C3 (degree = 36), FPR2 (degree = 36), CXCXL10 (degree = 35), C5AR1 (degree = 32), and IL1b (degree = 30). These genes could play a critical role in the PPI network.

### Co-expression Analysis of DElncRNAs and DEmRNAs

The correlation analysis of DElncRNAs and DEmRNAs was performed by calculating the Pearson correlation coefficients. In this study, the corrected *p*-value < 0.01 indicated that DElncRNAs were significantly correlated with DEmRNAs. The co-expression analysis results of DElncRNAs and DEmRNAs were listed in Table 1.

**TABLE 1:** Co-expression analysis results of DElncRNAs and DEmRNAs.

**DElncRNAs**	**Number of co-expressed DEmRNAs**	**DElncRNAs**	**Number of co-expressed DEmRNAs**
2010310C07Rik	205	Gm15569	70
AI506816	175	Gm39377	68
Gm19510	175	8030442B05Rik	65
C430002N11Rik	172	Gm16551	64
4930474M22Rik	149	Gm33100	64
E330032C10Rik	140	F630028O10Rik	54
A230001M10Rik	129	A730006G06Rik	49
Gm38403	115	Gm17830	48
C030013G03Rik	113	B230323A14Rik	47
Gm11827	113	Gm33680	42
H19	96	Gm29683	41
A530001N23Rik	94	Six3os1	37
4930572O13Rik	81	E230016K23Rik	36
C030029H02Rik	72	A330076C08Rik	15
Gm34567	71	AU023762	15

### Functional Similarity Analysis of DElncRNAs

Gene ontology and KEGG enrichment analyses of DElncRNAs were performed based on co-expressed DEmRNAs. Based on GO-BP results, we analyzed the functional similarity of these DElncRNAs by Gosemsim. As shown in Figure 2C, there were 24 DElncRNAs with similar functions, including 15 up- and 9 down-regulated lncRNAs. The thicker the line between the two DElncRNAs, the more similar the function between the two DElncRNAs.

### Identification of DEmiRNAs for ICH

**FIGURE 3:**
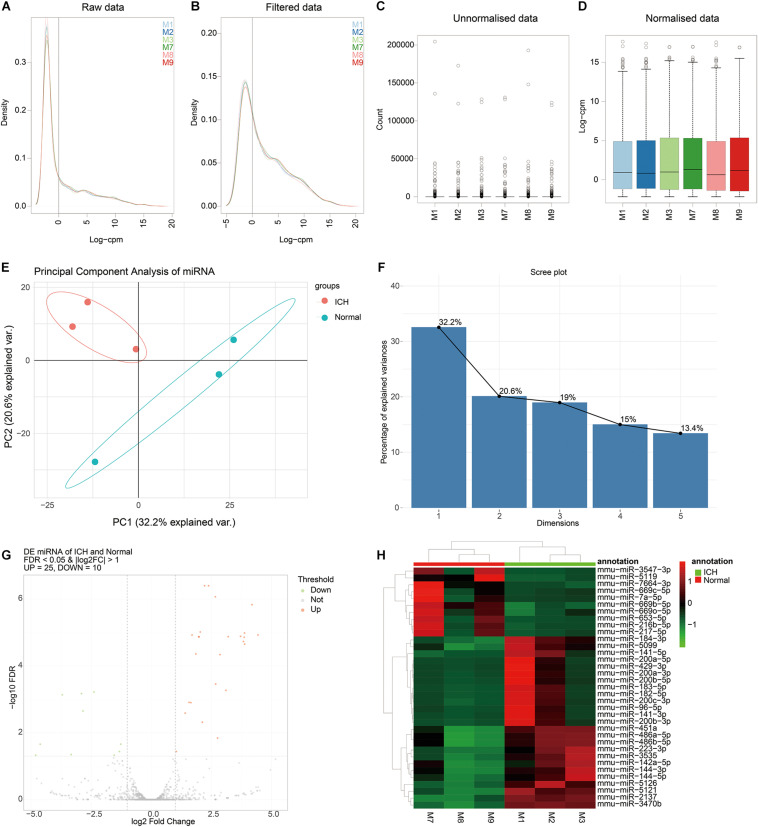
Identification of DEmiRNAs for ICH. **(A,B)** The data density distribution curve before or after removing low-expressed miRNAs. **(C,D)** The box plot showing the unnormalized or normalized data. **(E)** PCA results of miRNAs. **(F)** Scree plot of PCA. **(G)** Volcano plot showing DEmiRNAs between ICH and sham groups. **(H)** Hierarchical clustering analysis results depicting the expression patterns of DEmiRNAs between ICH and sham groups. M1-3 represents three ICH mouse models and M7-9 represent three sham operation mice. Red indicates up-regulated miRNAs and green indicates down-regulated miRNAs.

We further analyzed miRNA transcriptome for ICH. By data preprocessing, lowly expressed miRNAs were removed (Figures 3A,B). Then, the filtered data were normalized by TMM methods (Figures 3C,D). As shown in the PCA analysis results, the interpretation of the data by the two dimensions exceeded 50% (32.2% + 20.6%) in Figure 3E. Furthermore, from comp.2, the scree plot began to flatten, indicating that the two dimensions can well explain the characteristics of the data (Figure 3F).

After PCA, we performed differential expression analysis. The results showed that there were 25 up- and 10 down-regulated miRNAs between the ICH and sham groups (Figure 3G). As depicted in the hierarchical clustering analysis results, these DEmiRNAs could obviously distinguish the ICH group from the sham group (Figure 3H).

### Construction of a ceRNA Network for ICH

**FIGURE 4:**
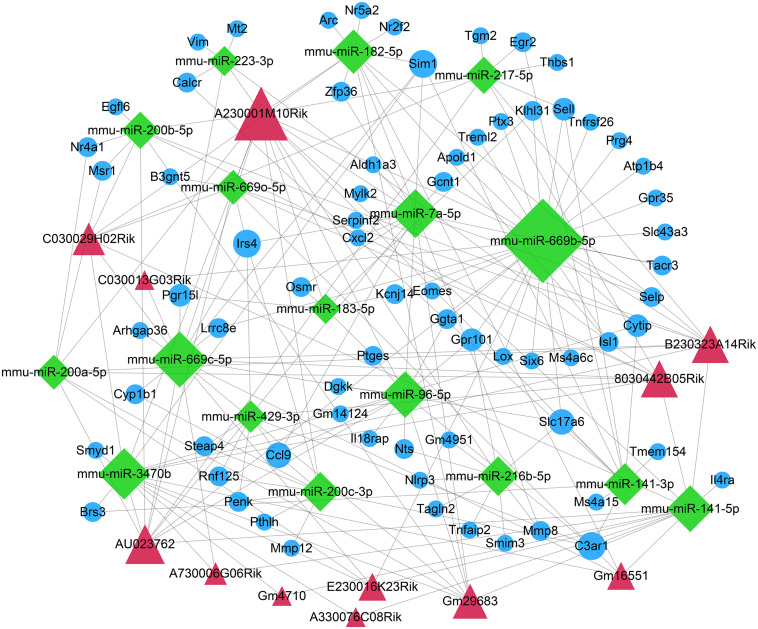
Construction of a ceRNA network for ICH. The red triangle represents the DElncRNAs, the green diamond represents DEmiRNAs, and the blue circle represents mRNAs. The node size indicates the degree.

The DEmiRNA–DEmRNA relationships were predicted using the miRWalk, Microt4, miRanda, mirbridge, miRDB, miRMap, miRNAMap, Pictar2, PITA RNA22, RNAhybrid, and Targetscan databases on miRWalk 2.0 platform. DEmiRNA–mRNA relationships supported by over seven databases were used for the construction of a ceRNA network. Furthermore, relationships between DElncRNAs and DEmiRNAs were predicted by the miRanda tool. In this study, if there were more than five binding sites, DElncRNA–DEmiRNA interactions were identified for further analysis. After integration of DEmiRNA–DEmRNA and DElncRNA–DEmiRNA relationships, we constructed a lncRNA–miRNA–mRNA ceRNA network (Figure 4). In the ceRNA network, there were 71 DEmRNAs, 17 DEmiRNAs, and 12 DElncRNAs. Furthermore, there were 78 DElncRNA–DEmiRNA relationships (Supplementary Table S1) and 119 DEmiRNA–DEmRNA relationships (Supplementary Table S2). The larger the node, the larger the degree, the more important the role of the node in the network.

### Identification of DEproteins for ICH and Functional Enrichment Analysis

**FIGURE 5:**
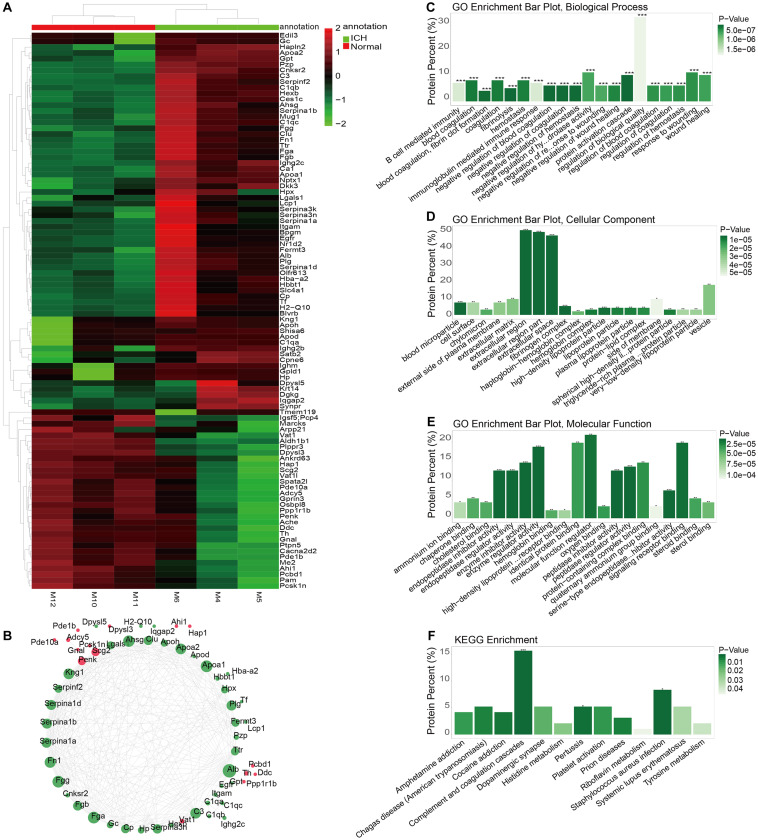
Identification of DEproteins for ICH and functional enrichment analysis. **(A)** Hierarchical clustering analysis results showing the expression patterns of 96 DEproteins between ICH and sham groups. M1-3 represents three ICH mouse models and M7-9 represent three sham operation mice. **(B)** Construction of a PPI network. Red represents up-regulation and green represents down-regulation. The circle size indicates the degree of the node in the PPI network. **(C)** GO-BP enrichment analysis results. **(D)** GO-CC enrichment analysis results. **(E)** GO-MF enrichment analysis results. **(F)** KEGG pathway enrichment analysis results. *p < 0.05; **p < 0.01; ***p < 0.001.

The whole proteomics resequencing of ICH model and sham groups was performed. Ninety six DEproteins were identified between ICH and sham groups. Hierarchical clustering analysis results suggested that these DEproteins could obviously distinguish ICH samples from sham samples (Figure 5A and Supplementary Table S3). To explore protein–protein interactions, we constructed a PPI network based on these DEproteins. In the PPI network, there were 58 nodes, including 15 highly expressed and 43 lowly expressed proteins (Figure 5B). The top ten nodes with the highest degree were as follows: Alb (degree = 31), Fga (degree = 26), Fgg (degree = 26), Apoa1 (degree = 26), Ahsg (degree = 25), Fn1 (degree = 24), Apoa2 (degree = 23), Kng1 (degree = 23), C3 (degree = 23), and Plg (degree = 21). These nodes could play important roles in the PPI network. We further explored the functions of these proteins. The top ten GO-BP enrichment analysis results were shown in Figure 5C, such as B cell mediated immunity, blood coagulation and hemostasis, and so on. As for GO-CC, these proteins were mainly enriched in extracellular regions, extracellular region parts and extracellular space (Figure 5D). Furthermore, these DEproteins were significantly associated with molecular function regulators, enzyme regulator activity, and signaling receptor binding (Figure 5E). KEGG enrichment analysis results showed that these DEproteins were mainly enriched in complement and coagulation cascades, pertussis, and staphylococcus aureus infection (Figure 5F). Thus, these proteins could be involved in various key biological processes and pathways.

### mRNA–Protein Correlation Analysis

**FIGURE 6:**
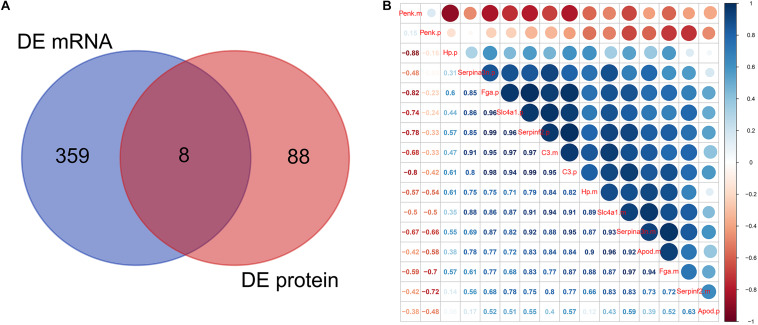
mRNA–protein correlation analysis. **(A)** Venn analysis of DEmRNAs and DEproteins. **(B)** Pearson correlation analysis results showing the correlation between DEmRNAs and DEproteins.

Venn analysis showed the intersections between DEmRNAs and DEproteins. We found that Fga, Hp, C3, Apod, Serpinf2, Penk, Serpina3n, and Slc4a1 were differentially expressed in ICH compared to sham groups at the mRNA and protein levels (Figure 6A). Pearson correlation analysis results show that there were high correlations between several DEmRNAs and DEproteins, indicating that there could be mutual regulatory relationships between these RNAs and proteins (Figure 6B). However, several DEproteins did not correlate with DEmRNAs, indicating that the two could have regulatory relationships for ICH.

### Validation of DEmRNAs and DEproteins for ICH

**FIGURE 7:**
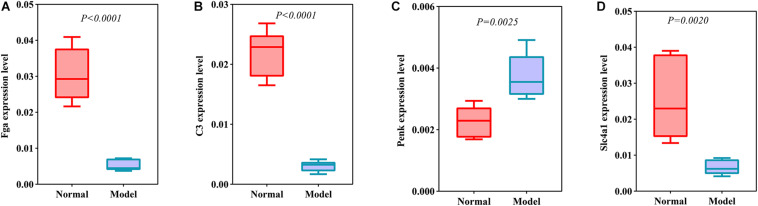
Validation of DEmRNAs in ipsilateral hemisphere tissues of ICH model and control groups using RT-qPCR. **(A)** Fga; **(B)** C3; **(C)** Penk; **(D)** Slc4a1.

**FIGURE 8:**
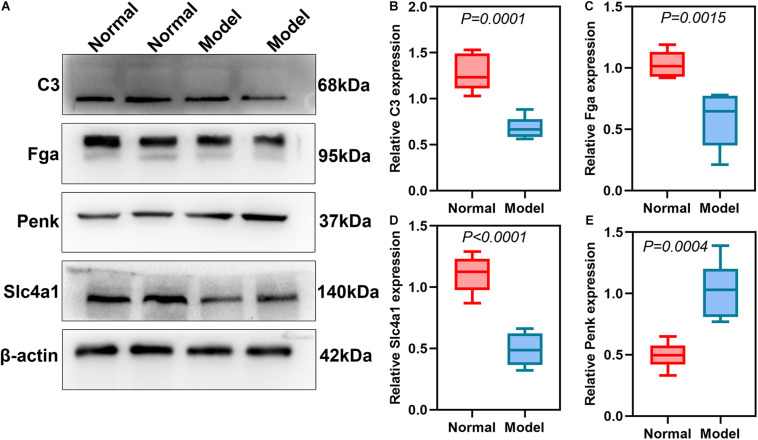
Validation of DEproteins in ipsilateral hemisphere tissues of ICH model and control groups using RT-qPCR. **(A)** Representative images of western blot results. **(B)** C3; **(C)** Fga; **(D)** Slc4a1; **(E)** Penk.

DEmRNAs and DEproteins were validated in ipsilateral hemisphere tissues of ICH model and control groups using RT-qPCR and western blot. Our RT-qPCR confirmed that Fga (*p* < 0.0001; Figure 7A), C3 (*p* < 0.0001; Figure 7B), Penk (*p* = 0.0025; Figure 7C), and Slc4a1 (*p* = 0.0020; Figure 7D) were differentially expressed between ICH and control groups. Among them, Fga, C3, and Slc4a1 mRNAs were markedly down-regulated in ICH than controls. Furthermore, we examined the expression of Fga, C3, Penk, and Slc4a1 proteins in ipsilateral hemisphere tissues by western blot (Figure 8A). Consistently, the data showed that C3 (*p* = 0.0001; Figure 8B), Fga (*p* = 0.0015; Figure 8C), and Slc4a1 (*p* < 0.0001; Figure 8D) proteins were lowly expressed in ICH compared to control groups. Penk (*p* = 0.0004; Figure 8E) exhibited higher expression levels in the ICH model group than the control group.

## Discussion

Currently, research on the treatment of ICH mainly focuses on the clinical aspects of therapeutic intervention, however, the molecular mechanism of ICH is still unclear (Yin et al., 2017). Therefore, the identification of therapeutic targets for ICH can improve the treatment strategies for ICH. In this study, we conducted a collagenase VII-induced ICH model. The whole transcriptome resequencing analysis was performed between ICH model and sham groups. Increasing evidence has confirmed that lncRNA is involved in brain function and neurological diseases (Jia et al., 2018; Zhang and Wang, 2019; Zhang et al., 2019). However, the expression characteristics of lncRNA after ICH remain to be elucidated. In this study, we analyzed the expression patterns of lncRNAs between collagenase VII-induced ICH mouse model and sham operation mice. After preprocessing, we identified 31 DElncRNAs in the ICH model compared to sham group, including 11 up- and 20 down-regulated lncRNAs. Furthermore, 367 DEmRNAs were obtained between ICH and sham groups, including 306 up- and 64 down-regulated mRNAs. These DElncRNAs and DEmRNAs can obviously distinguish ICH from sham operation groups, which could be involved in the pathological processes of ICH.

We further explored the potential functions of DEmRNAs by GO and KEGG functional enrichment analyses. We found that these DEmRNAs were mainly enriched in inflammation responses. It has been confirmed that inflammation plays an important role in the brain injury after ICH (Zhou et al., 2014). Furthermore, the mechanism of ICH is closely related to the infiltration of inflammatory cells (Lan et al., 2019). However, few effective treatments have been found so far. Our GO enrichment analysis results showed that these DEmRNAs were in association with leukocytes, neutrophils, granulocytes, and so on. It has been confirmed that acute leukocytosis responds to ICH (Morotti et al., 2016). Neutrophil infiltration as a therapeutic target can aggravate brain damage after ICH (Zhao et al., 2017). A clinical study has reported that the neutrophil-to-lymphocyte ratio can predict acute ICH up to 3 months in advance (Lattanzi et al., 2016). Moreover, granulocyte colony-stimulating factor can alleviate neurological function and angiogenesis in ICH rat model (Liang et al., 2018). KEGG pathway results showed that these DEmRNAs were also mainly enriched in immune-related pathways such as IL-17, TNF, and JAK-STAT signaling pathways. Pro-inflammatory cytokines IL-17 and TNF-α are elevated in the serum of patients with neurodegenerative diseases, including ICH (Yang and Shao, 2016; Chaudhry et al., 2017). A previous study has reported that osteopontin could attenuate inflammatory response by JAK2/STAT1 in ICH hyperglycemic rat models (Gong et al., 2018). Based on previous findings, after the onset of ICH, cytokines released by various inflammatory cells are involved in secondary brain damage caused by ICH. There are many potential genes in the pathological processes of ICH. Thus, these DEmRNAs might participate in inflammation after ICH, which could be considered as potential therapeutic targets.

It is critical to explore the interactions between DEmRNAs and understand their biological significance. In our study, we constructed a PPI network based on DEmRNAs for ICH, including 151 up- and 33 down-related genes. The nodes with the highest degree could become potential hub genes, including IL6, TNF, CXCR2, CXCL1, CXCL2, C3, FPR2, CXCXL10, C5AR1, and IL1b. Most of them are inflammatory cytokines, suggesting that immunity plays an important role in the pathological processes of ICH. Pearson correlation analysis was used to explore correlation DElncRNAs and DEmRNAs. The functions of DElncRNAs were predicted by functional enrichment analysis based on their co-expressed DEmRNAs. Furthermore, we found that 24 DElncRNAs could possess similar functions by Gosemsim, including 15 up- and 9 down-regulated lncRNAs. In line with a previous study, lncRNA H19 is up-regulated in the ICH model compared to controls (Kim et al., 2019). These DElncRNAs require further exploration.

We further analyzed miRNA transcriptome for ICH and identified 25 up- and 10 down-regulated miRNAs between ICH and sham groups. These DEmiRNAs had a significant difference between the ICH group and sham group. lncRNA can respond to miRNAs with specific miRNA response elements in the 3′UTR region, and then represses the expression of miRNA targets (Tay et al., 2014). In our study, we predicted the DElncRNA–DEmiRNA and DEmiRNA–DEmRNA relationship pairs. After integration of DEmiRNA–DEmRNA and DElncRNA–DEmiRNA relationships, we constructed a lncRNA–miRNA–mRNA ceRNA network. The regulatory mechanism of the ceRNA network in ICH remains unknown (Dou et al., 2020). Our findings proposed the ceRNA network of ICH that deepened the understanding of molecular mechanisms of ICH.

In this study, 96 DEproteins were identified between ICH and sham groups. As depicted in the PPI network, Alb, Fga, Fgg, Apoa1, Ahsg, Fn1, Apoa2, Kng1, C3, and Plg could become potential hub proteins, which could play important roles in ICH. These DEproteins were significantly associated with B cell mediated immunity, blood coagulation, and hemostasis, indicating that these DEproteins could be involved in ICH. Our results showed that Fga, Hp, C3, Apod, Serpinf2, Penk, Serpina3n, and Slc4a1 were differentially expressed in ICH at the mRNA and protein levels. A clinical study found that FGA Thr312Ala polymorphism could affect the risk of ICH in Polish participants (Jagiella et al., 2014). A study has confirmed that C3 is in association with the risk of ICH (Jiang et al., 2015). Furthermore, it can cause brain injury induced by ICH (Yang et al., 2006). After validation using RT-qPCR and western blot, our study confirmed that C3, Fga, and Slc4a1 were lowly expressed and Penk was highly expressed in ICH compared to control groups. These markers could be involved in the progression of ICH. More experiments should be presented to validate their biological functions in ICH.

## Conclusion

In this study, we constructed an ICH model. By comprehensively analyzing transcriptome resequencing and quantitative proteomic analyses, we identified ICH-related DE-RNAs and proteins and potential molecular mechanisms of ICH, which are worthy of further exploration.

## Data Availability Statement

The original contributions presented in the study are included in the article/Supplementary Material, further inquiries can be directed to the corresponding author/s.

## Ethics Statement

The animal study was reviewed and approved by Animal Ethics Committee of Zunyi Medical University.

## Author Contributions

SY and FC conducted, conceived of, and designed the experiments, and wrote the manuscript. YG did the MRI scan. HZ and QZ analyzed the data. YF, JS, and QL built the models. All authors read and approved the final manuscript.

## Conflict of Interest

The authors declare that the research was conducted in the absence of any commercial or financial relationships that could be construed as a potential conflict of interest.

## Supplementary Material

Supplementary Figure 1 T2-weighted images of ICH mouse models.

Supplementary Table 1 Seventy eight DElncRNA–DEmiRNA relationships in the ceRNA network.

Supplementary Table 2 One hundred and nineteen DEmiRNA–DEmRNA relationships in the ceRNA network.

Supplementary Table 3 Ninety six DEproteins between ICH and sham groups.

## Abbreviations

ICH: intracerebral hemorrhage
DElncRNAs: differentially expressed lncRNAs (DElncRNAs)
DEmRNAs: differentially expressed mRNAs
DEmiRNAs: differentially expressed miRNAs
DEproteins: differentially expressed proteins
PPI: protein–protein interaction
ceRNA: competing endogenous RNA
ncRNAs: non-coding RNAs
GO: gene ontology
KEGG: Kyoto Encyclopedia of Genes and Genomes (KEGG)
CC: cellular component
MF: molecular function
BP: biological process.
