# Supplementary figures and images for: Integration of Transcriptome Resequencing and Quantitative Proteomics Analyses of Collagenase VII-Induced Intracerebral Hemorrhage in Mice
Source: Front Genet. 2020 Dec 17;11:551065. doi: 10.3389/fgene.2020.551065 (PMC7793737; doi:10.3389/fgene.2020.551065)

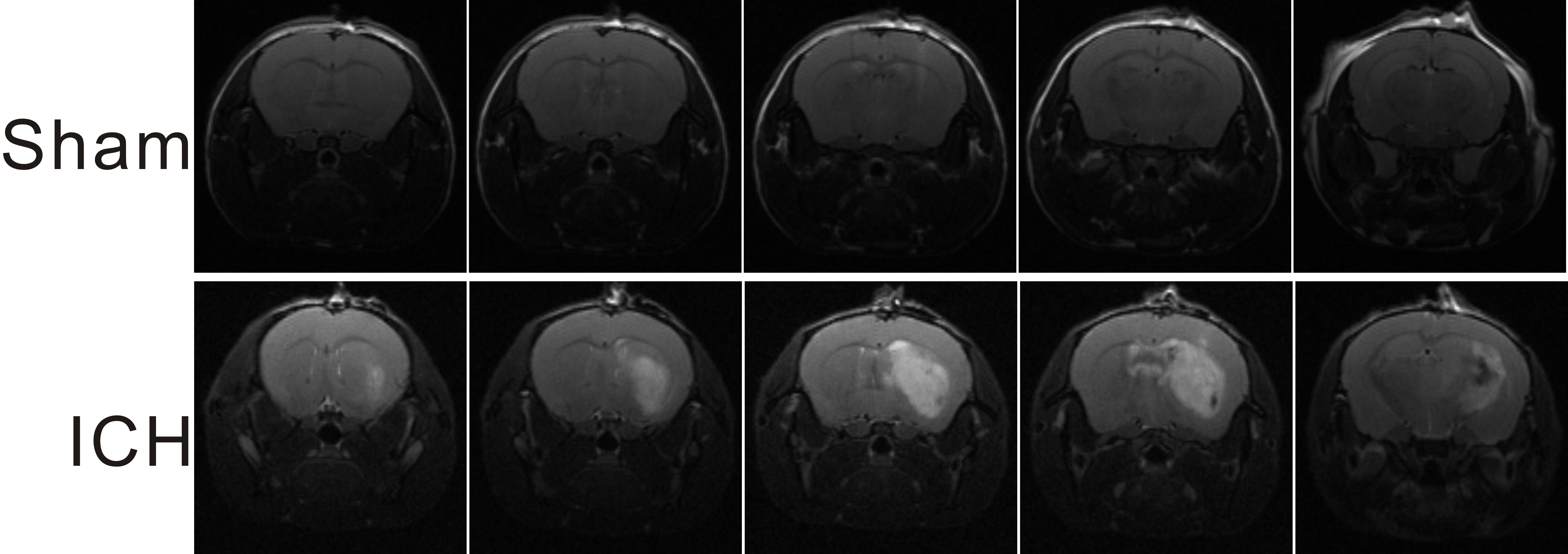

Supplement: Supplementary Figure 1 — T2-weighted images of ICH mouse models. [file Image_1.TIF]
